# Desiring Neoliberalism

**DOI:** 10.1007/s13178-016-0257-6

**Published:** 2016-10-05

**Authors:** Gundula Ludwig

**Affiliations:** Department of Political Science, University of Vienna, Universitätsstrasse 7/2, 1010 Vienna, Austria

**Keywords:** Neoliberalism, Sexual politics, Governmentality, Statehood, Apparatus of sexuality

## Abstract

The paper is based on the premise that neoliberalism is a political rationality that is not only anti-social but also requires an anti-democratic and violent form of statehood. However, neoliberalism is not solely based on coercion and force, but paradoxically also on consensus. This consensus is not least organized through its flexibilized and pluralized sexual politics. By focussing on sexual politics in Germany’s capital Berlin, the paper highlights that the flexibilization of the apparatus of sexuality is not merely a side effect of neoliberalism but a constitutive element of neoliberal governmentality that is deployed to legitimate an anti-democratic and violent neoliberal state. Neoliberalism uses the promise of sexual tolerance, flexibility, and pluralism in order to fulfill its anti-social, anti-democratic, and violent agenda. Furthermore, it is argued that neoliberal sexual politics require a rethinking of the concept of heteronormativity. Here, I propose to recast heteronormativity as heteronormalization.

In July 2015, as I began writing this article, the arguments I had set out to make were vividly apparent as I walked through the streets of the German capital Berlin. At that same time that the government buildings in Berlin were covered with rainbow flags in the wake of the city’s annual Christopher Street Day celebrations, the papers were filled with news on two ‘key problems’ the European Union and Germany were currently facing: the outcome of the referendum on the European austerity measures in Greece; and the situation of refugees in the European Union. Notably, the discourse around the latter was framed using terms such as ‘refugee problem’ indicating ‘anxiety’ that ‘too many’ refugees would enter the European Union. The simultaneous occurrence of the metropolis promoting itself as gay-friendly by cloaking official buildings with rainbow flags and Germany’s role as key actor in working toward implementing restrictive authoritarian policies as a ‘response’ to both the current economic crisis and the situation of refugees in the European Union is a vivid example of the current neoliberal political and social order. Without a doubt, the emergence of neoliberalism in Western societies has led to an increase in more harsh political regulations of class relations and migration regimes. Examples found in the neoliberal program include the cancellation of welfare programs and public funding for social services, the privatization of schools, hospitals and other formerly publically run institutions, curtailing rights for workers and trade unions, cutbacks on state support for refugees, cuts in state support for NGOs supporting refugees, and a boom in discourses that there are ‘too many refugees’ for Western nations to deal with.

The picture changes, however, when we take the regulation of sexuality into consideration. Here, neoliberalism appears to be based on a political rationality that is geared toward increasing freedom and tolerance. The emergence of neoliberalism in Western nation states has coincided with more liberal, tolerant, inclusive, and diverse sexual politics. Under neoliberalism rigid heteronormative politics have become flexibilized, same-sex lifestyles are no longer criminalized and pathologized, and same-sex partnerships have gained legal recognition. These legislative changes have also gone hand in hand with a liberalization of social attitudes toward certain non-heterosexual lifestyles—in particular white, non-migrant, non-muslim, middle and upper class lesbians and gays. Against this background, Lisa Duggan has rightly described neoliberal sexual politics as “homonormative” (2003), Jasbir Puar as expression of “homonationalism” (2007) and Chandan Reddy has argued that the state’s desire for “gays’ and lesbians’ desire for recognition” (Reddy 2011: 193) is a key element of the neoliberal state.

These two faces of neoliberalism provide the point of departure for my article. The aim of the text is twofold: First, I argue that neoliberalism does not only *effect* sexual politics. Rather, the flexibilization of sexual politics needs to be understood as intrinsic and productive element of neoliberal governmentality that is deployed to legitimate an anti-democratic and violent neoliberal state. Second, I argue that neoliberal sexual politics require a rethinking of the concept of heteronormativity. Here, I propose to recast heteronormativity as heteronormalization. The article focuses on neoliberal statehood in Germany in particular, although some of the aspects of neoliberal statehood and the role of sexual politics for neoliberal governmentality and statehood may also apply to other neoliberal nation states.

## State Power, Sexual Politics, and Capitalism

Let me introduce the theoretical frame of my argumentation by briefly referring to Michel Foucault’s work on the relation between state and sexuality in capitalist societies. Foucault has taught us that neither sexuality nor sexual identity are naturally given. Instead, they are an effect of the apparatus of sexuality; sexuality is a counterpart of biopower, a form of power that “bent on generating forces, making them grow, and ordering them” (Foucault 1990: 136). Sexual politics are a means of obtaining access to the life of an individual body and to the lives of the entire population. For Foucault, sexuality is a “concern of the state” (Foucault 1990: 116): the state *governs* its citizens’ sexuality, reproductive activities, birth and marriages practices by inciting self-activities in the subjects. Thus, sexual politics are not solely forced upon the subjects. Subjects *conduct and govern themselves* in their sexual behavior and body politics by applying technologies of power to themselves.

Foucault illustrates that capitalism requires sexual politics and biopower (Foucault 1990: 140). However, he also reminds us that the relationship between capitalism, biopower, and sexuality is not only repressive and functionalist. Capitalism not only requires repression, force, and discipline but also technologies of biopower that incite the population to constantly enlarge and optimize its forces and its capabilities (Foucault 1990: 137). Foucault not only relates biopower to capitalism but also to racism in its “modern, ‘biologizing, statist form” (Foucault 1990: 149): The biologizing construct of ‘race’ introduces a censorship between those, whose lives should be optimized and proliferated and those whose lives should not. Given that biopower is a form of power that aims at optimizing some lives, consequently, the death of some ‘othered’ others is required in order to optimize some lives (Foucault 1997).

Let me point out one last Foucauldian argument that is necessary to understand the interplay between sexuality and the state in capitalist societies: In his lectures on governmentality, Foucault rejects the assumption that the modern Western state can be grasped as a universal, given entity—as in liberal theory. On the contrary, Foucault argues that the “state is a practice” (Foucault 2007: 277). Political rationalities guide social practices and enable the existence of the modern state. Only when the state is “called for, desired, coveted, feared, rejected, loved, and hated” (Foucault 2007: 247) is a historic form of state brought into existence. Consequently, the state needs to be built upon the consensus among the majority of its subjects and a desire to be part of a historically specific statehood, which these subjects then act upon and integrate into their everyday practices.

In the following, I take these Foucauldian perspectives on capitalism, state power and sexual politics as analytical instrument to approach the interplay of sexual politics and neoliberalism.

## The Flexibilization of the Apparatus of Sexuality

In 2001, Germany introduced a same-sex-partnership law; since 2005 same-sex partners can adopt their partner’s children, and in 2014 lesbian and gay couples received the right to adopt the adopted child of their partner. Joint adoption of a child by same-sex partners is not permitted currently, but the ongoing political and legal debates today indicate that in the near future the Federal Constitutional Court is likely to rule against this existing discrimination. In 2013, the Federal Constitutional Court ruled that excluding same-sex partnerships from the “Ehegattensplitting”, a tax system in Germany which spouses each pay income tax on half the total of their combined incomes, unconstitutional.

These legal changes were accompanied by a political rhetoric which frames diversity, plurality and tolerance as hallmarks of Germany’s national identity—an image that is in particular fostered by the City of Berlin that presents itself as “rainbow capital” on its official webpage (www.berlin.de)—because it has one of the biggest lesbian, gay and trans communities and is a magnet for queer tourism (see https://www.berlin.de/lb/ads/schwerpunkte/lsbti/).^1^ A core strategy for the Senate of the City of Berlin as ‘rainbow capital’ was to propose and implement a campaign called “Berlin Advocates Self-Determination and Acceptance of Sexual Diversity” (“Berlin tritt ein für Selbstbestimmung und Akzeptanz sexueller Vielfalt”) in 2010. The premise of the campaign is that Berlin is a metropolis of “openness” (Senate of Berlin 2010: 1, author’s translation) and, as such, also a “city of diverse cultures, conceptions and ways of living” (Senate of Berlin 2010: 1, author’s translation). It ascribes to “cultural diversity and a variety of sexual orientations, identities and individual life concepts” (Senate of Berlin 2010: 1, author’s translation). The campaign aims to introduce sexual diversity into education and administration, to advance legal equality and initiate dialogues with civil society institutions to increase sexual tolerance and sexual diversity.

Not only in the realm of laws and politics sexual politics have fundamentally changed: the economy has also discovered diversity as a promising factor for success. Lesbians and in particular gays have been identified as an important consumer group and thus been featured as subjects and directly addressed in commercials (for an analysis of advertisements aimed at queers, see Engel 2009). Furthermore, many companies have started to promote diversity and plurality as guarantee for creativity, innovation and success. In 2006, the “Charter of Diversity” (“Charta der Vielfalt”) was laid out by a number of large companies including Daimler, BP Europa SE, Deutsche Bank and Deutsche Telekom with the aim of bringing more awareness and openness for diversity into companies so that “all colleagues irrespective of gender, nationality, ethnic background, religion or worldview, disability, age, and sexual preference and identity” (Charta für Vielfalt 2015) feel appreciated. Their premise is that an “atmosphere of acceptance and mutual trust” (Vielfalt 2015) leads to economic success. Since the founding year, more than 2250 companies and public institutions have signed the charter. They also host the annual “German’s Diversity Day” (“Deutscher Diversity-Tag”) where in 2015 over 850 events took place across Germany.

These examples show that along with the emergence of neoliberalism the apparatus of sexuality also underwent a transformation. ‘Normal’ sexuality is no longer defined solely on its relation to matrimonial reproduction. Certain guises of homosexuality have been included in what is considered ‘normal’. Ways of living same-sex desire and lifestyles that resemble the heterosexual ideal of the monogamous, faithful couple and the ideal of the heteronormative family are now legally recognized and socially tolerated. Within neoliberal societies, same-sex partnerships and families also count as protectable and “deserving of social support” (Schwesig 2014: 4, author’s translation) as the Minister for Family, Senior Citizens, Women and Youth, Maria Schwesig, wrote in her preface to the brochure “Rainbow Families – unexceptional but different” (“Regenbogenfamilien – alltäglich und doch anders”) by the Lesbian and Gay Association Germany (“Lesben- und Schwulenverband in Deutschland”/LSVD). Finally, the ideal of sexual self-determination has come in to replace the ideal of sexual self-control. In the name of difference, plurality, and tolerance, the apparatus of sexuality has become more open and flexible.

From a Foucauldian perspective, the flexibilization of the apparatus of sexuality and heteronormativity cannot be read as an outcome of—or deduced from—neoliberal capitalism. Following Foucault’s argument, in order to understand the relationship between power and sexuality, it is not only necessary to consider the requirements of the mode of the production, but we must also sharpen the focus on the interplay between state power, population and sexuality under capitalism. For this reason, rather than asking merely how neoliberalism impacts current sexual politics, I explore how sexual politics enable a specific form of governing and thus, technologies of power that not only operate through “means of deduction” (Foucault 1990: 136) but as technologies of power that are “bent on generating forces, making them grow, and ordering them” (Foucault 1990: 136). Or respectively: how sexual politics help to constitute neoliberal subjects, society and statehood. Hence, neoliberalism entails much more than integrating gays and lesbians into capitalism by rendering them too as consumers, as some discussions of neoliberalism and sexuality have suggested (Chasin 2000; Evans 1993). Instead, neoliberalism calls for a fundamental restructuring of sexual politics so that individuals and the population can be made governable as well as govern themselves according to neoliberal ‘regimes of truth’. In order to illustrate the linkage between neoliberal governmentality and sexual politics let me focus on four dimensions.

## The Flexibilization of Heteronormativity as an Element of Neoliberal Governmentality

First, the flexibilization of the apparatus of sexuality fosters neoliberalism’s anti-naturalism. Neoliberal governmentality is built upon an anti-naturalistic political ontology since it neither grasps the market nor the existence of *homines oeconomici* as naturally given but as artificially produced, as Foucault has highlighted in his analysis of neoliberal governmentality (Foucault 2008: 31; see also Oksala 2011: 477). In contemporary neoliberal sexual politics, we also find anti-naturalism as a motif. Not only is the neoliberal apparatus of sexuality no longer based on the rigid assumption that heterosexuality is naturally given but also the reference to ‘naturalism’ has lost significance in governing sexualities. Integrating (some) lesbian and gay lifestyles into what is considered acceptable and legally and socially protectable is based on the discursive logic of locating lesbian and gay lifestyles within the same continuum of normality as heterosexuality. The campaign “It’s no different in our homes” (“Bei uns geht’s auch nicht anders zu”) promoting ‘rainbow families’, which was part of the “Campaign for Self-Determination and Sexual Diversity” that LSVD among others supported, even includes the sameness of ‘rainbow’ and heterosexual families in its title (LSVD 2015). Similarly, in the preface to LSVD’s brochure on ‘rainbow families’, Maria Schwesig, Minister for Family, Senior Citizens, Women and Youth draws on this same logic by pointing to a continuum between heterosexual and same-sex parents (Schwesig 2014). These examples show that differences “are no longer seen as essential or absolute ‘otherness’ but rather as particularity, hybridity, and the products of individual practices in need of continuous refinement” (Engel 2011: 75). In neoliberal societies difference is not strictly derived from a given norm and used to identify ‘deviant’ or non-natural beings. On the contrary, due to a blurring of the distinction between ‘normal’ and ‘non-normal’ subjects and to the increasing importance of individualization, “everybody is expected to find ways of expressing difference as particularity and specialness” (Engel 2011: 75).

Second, by converting inequality into disparity neoliberal sexual politics help to constitute and reproduce a diverse society based on competition. Foucault has argued that neoliberal governmentality replaces the former liberal principle of exchange with the principle of competition as a key strategy of the capitalist economy (Foucault 2008: 118). Competition is the premise for neoliberal society and, as such, the state is called upon to secure the preconditions for competition. Based on their own premise of an anti-naturalistic political ontology, in neoliberalism’s view, competition does not follow—in contrast to liberalism’s interest in free exchange—from a naturally given will, instinct or appetite, but it needs to be politically constituted. Neoliberalism requires that the state organizes—or “artificially constructs” (Foucault 2008: 120)—a formal structure so that competition can develop. In this vein, Foucault points out that whereas “governmental intervention must be light at the level of economic processes”, they must “be heavy when it is a matter of this set of technical, scientific, legal, geographic, let’s say, broadly, social factors” (Foucault 2008: 141). Thus, while the principle of competition requires only ‘light’ interventions on the level of the market, expanding the principle of competition demands state interventions that produce and acknowledge differences and disparity. Unlike Fordist society and its mode of production, neoliberal society and its mode of production are not built upon standardized forms and homogeneity but rather on flexible modes of production, consumption and living (Foucault 2008: 259). The economization of the entire society encourages its subjects to arrange and manage their lives individually rather than to orientate themselves in their everyday lives in a way that is based on a priori given, rigid norms.

One social factor the state governs is sexuality. Recognizing and appreciating (certain) non-heterosexual sexualities fosters a disparate and unequal society where the disparities and inequalities are not viewed as problematic, but are framed as proof of tolerance, plurality and freedom. These paradigms of individuality and diversity are not only promoted by campaigns such as “Berlin Advocates Self-Determination and Acceptance of Sexual Diversity” and in the “Charter of Diversity”. These campaigns focus on ‘sexual orientation’ and ‘sexual identity’ because they are framed as one of the “core dimensions” (Charta für Vielfalt 2011, author’s translation) of individuals. At the same time, the charter closely links diversity to economic success: “We have come to realize that we can only be successful economically if we acknowledge and leverage the existing diversity” (Charta für Vielfalt 2015). Recognizing sexual pluralities can thus function as an important tool in rendering the whole of society more diverse and lends itself to constituting a social framework in which competition is a core principle.

Against this background, (some) gay and lesbian ‘lifestyles’ can be seen as ‘prototypes’ of a neoliberal society. Antke Engel describes the process of integrating (some) gay and lesbian lifestyles into neoliberal forms of normality as “projective integration” (Engel 2011). ‘Projective integration’ is characterized by a positive and affirmative position toward differences viewed as employable cultural capital. Imagining a ‘homosexual lifestyle’ in which subjects are flexible, dynamic and self-determined configures gays and lesbians as neoliberal role models, in contrast to the Fordist heterosexual couple that organized their lives based on standardization, inflexibility and predictability. “Projective integration fulfills a double function: normalized subjects can project their desires onto images of difference, while dissident or marginalized subjects enjoy inhabiting an avant-garde position” (Engel 2011: 74). Positive images of lesbian and gay ‘lifestyles’ “act as screens of projection that stand in for individuality, flexibility, and above all, for the ability to manage the contradictory demands of late-modern life” (Engel 2011: 74). In these discourses that Engel describes as ‘projective integration’ the ‘promise of happiness’ (Ahmed 2010) operates as a crucial technology of power: It not only helps to integrate and assimilate lesbian and gay lifestyles but at the same time it stabilizes heteronormative paradigms of happiness precisely through the ‘projective integration’ of (some) queer lifestyles (see also Berlant 2011; Nay 2014). These heteronormative affective promises are both, a “process of hegemonic consensus production” and a “modernization of heteronormativity” (Engel 2011: 75). They strengthen a “society that is not orientated toward the commodity and the uniformity of the commodity, but toward the multiplicity and differentiation of enterprises” (Foucault 2008: 149). ‘Promises of happiness’ operate as neoliberal technology of power that support a capitalist mode of production that is built upon plural, flexible and diverse enterprises where each of them promises individualized satisfaction and individualized ‘well-being’ (Aschoff 2015; Davies 2015).

Third, sexuality is a crucial construction in making the subjects governable through their interpellation as entrepreneurial selves. Neoliberal governmentality redefines the *homo oeconomicus*: It is not merely a partner who engages in an economic exchange, but an entrepreneur of oneself (Foucault 2008: 226). As a consequence of the marketization of the entire society, the model of the market is applied to “every social actor in general” (Foucault 2008: 268). The individual should consider her- or himself as a “sort of permanent and multiple enterprise” (Foucault 2008: 241). Privatization and individualization encourage neoliberal subjects to conduct themselves as entrepreneurial selves. These neoliberal core strategies are promoted in the name of freedom (Foucault 2008: 63). An important element in governing the subjects as entrepreneurial selves is the “cult of being special” (Bröckling 2000: 158, author’s translation). The entrepreneurial self implies self-conduct, which enables a person to develop his or her own specificity and individuality. Neoliberalism offers the promise of being able to be one’s true self, which is framed as the ultimate freedom. These promises of individuality and freedom are closely linked to sexuality. Enabling the neoliberal subject to have a self-determined sexuality means giving them the possibility to obtain complete self-realization. The campaign “Berlin Advocates Self-Determination and Acceptance of Sexual Diversity” clearly links ‘self-determination’ to sexuality (as the title already points at) claiming that self-determination requires sexual diversity.

Thus, also in neoliberalism, sexuality remains a crucial construct that renders subjects governable. What has changed in the governing technology of neoliberalism, however, is that the sole aim of sexuality is no longer reproduction and rigid self-control. Neoliberal subjects are no longer primarily governed by an ideal of a strictly heterosexual and self-controlled sexuality. Instead, subjects are asked to find their ‘own’ self-determined sexuality within a plurality of sexualities, to express it, and to allow their choice to be recognized by a tolerant and diverse neoliberal society. Since sexuality is viewed as inner and intimate truth also in the neoliberalized apparatus of sexuality (as both the campaign “Berlin Advocates Self-Determination and Acceptance of Sexual Diversity” and the “Charter of Diversity” argue), this ultimately ‘liberated’ inner sexuality makes subjects governable and helps to turn them into entrepreneurial selves who are supposed to consider themselves as ‘free’ subjects.

Precisely because sexuality is constructed as a force that individualizes subjects, it is a powerful tool that aids in anchoring “neoliberalism’s key terms” “*privatization* and *personal responsibility*” (Duggan 2003: 12) in people’s everyday lives. The entrepreneurial *homo oeconomicus* is not only responsible for his or her own economic fortune but also for his or her ‘own’ and ‘specific’ sexuality, desire, reproduction and family. Neoliberal subjects conduct themselves as *homines oeconomici*—not because they are forced to—but because they also see perspectives for themselves within this mode of existence. Framing sexual plurality as means of realizing a society full of self-determined, free and self-responsible subjects can then be viewed as technology of power that helps render the entrepreneurial model “a model of social relations and of existence itself, a form of relationship of the individual to himself, time, those around him, the group, and the family” (Foucault 2008: 242). Advocating sexual self-determination also fosters the image of the subject as *homo oeconomicus* because both emphasize individual freedom and self-responsibility as key aspects of a good life.

Fourth, the emergence of what Lisa Duggan has described as “homonormativity” (Duggan 2002: 179) advances the anti-social attitude of neoliberal governmentality. In neoliberal societies, the social is dismantled in the name of privatization and individual freedom. Neoliberal governmentality promotes individuality and personal responsibility, presenting them as hallmarks of freedom and at the same time it frames social responsibility as the epitome of dependency and paternalism and as a threat freedom. These anti-social assumptions take on material forms in the dismantling of the welfare state, the marketization of social relations and social institutions, and the privatization of social risks (Foucault 2008: 144).

Homonormative politics buy into neoliberalism’s anti-social program: the legal and social recognition of same-sex partnerships and ‘rainbow families’ strengthens an ideal of an anti-social society by expanding marriage and its underlying ideals of privatizing social responsibilities to also encompass non-heterosexual partnerships (Duggan 2002; Warner 1999). As Duggan points out, homonormative politics link homosexuality to “domesticity and consumption” (Duggan 2002: 179). The legal institution of same-sex partnerships that can be viewed as the main politics of homonormativity reiterates the heteronormative ideal of a privatized, domesticized mode of existence. It also reinforces (neo-)liberal norms, which dictate the private organization of care, family and social reproduction. By doing so, homonormative ways of living support and (re-)produce the neoliberal ideals of privatization, individual freedom and independence from society and the state.

Furthermore, homonormativity also needs to be seen as a counterpart to the neoliberal moves to dismantle the welfare state. By including same-sex partnerships in the neoliberal project of privatizing social issues, in neoliberal societies all family constellations—not only heterosexual families—are asked to compensate for the dismantling of the welfare state. Neoliberal governmentality also invites same-sex partnerships and ‘rainbow families’ to perform the outsourced tasks within their own private spheres. The political campaigns as well as the legal reforms that aim to normalize ‘rainbow families’ seek to expand the group of people addressed as being responsible for performing the tasks of social reproduction within the private realm. Equating rainbow and heterosexual family arrangements also renders ‘rainbow families’ equal in terms of their obligation to organize social reproduction in the private realm. Volker Woltersdorff thus concludes: “social de-solidarization is the historical precondition of the state recognition of some non-heterosexual ways of living” (Woltersdorff 2004: 146, author’s translation).

The four dimensions demonstrate that the flexibilization of the apparatus of sexuality not only corresponds with neoliberal governmentality, but needs to be conceived as productive element of neoliberal governmentality. The transformation of the apparatus of sexuality helps govern subjects in a way that enables them to accept neoliberal ‘regimes of truths’ and integrate them into their everyday lives. This includes viewing self-determination, privacy and freedom as desirable values, favoring ‘diversity’ and ‘plurality’ over solidarity, equality and collectivity, considering self-determination and self-responsibility as key elements for one’s pursuit of happiness. Governing sexuality in the name of individual freedom, tolerance and diversity helps to constitute neoliberal subjects and a neoliberal population where individuals consider themselves as free entrepreneurial selves and where society is based on marketization and competition as its principle structures. Thus, the transformation of the apparatus of sexuality is not merely a side effect of neoliberalism. The diversification and pluralization of what counts as a ‘normal’ or ‘acceptable’ form of sexuality, desire, partnership or family imply that individuals and the population are governed in a way that helps them integrate neoliberal governmentality into their everyday practices and behavior. The flexibilization of the apparatus of sexuality helps produce a social reality that presupposes the existence of neoliberal governmentality.

## Heteronormalization as Neoliberal Technology of Power

What implications does the neoliberalization of the apparatus of sexuality have for conceptualizing heteronormativity?

The premise of flexibilizing the apparatus of sexuality is that lesbians and gays are integrated into the continuum of normality on the basis that they are ‘like heterosexuals’. This logic of sameness surfaces in moves to legitimize legal equality such as extending tax breaks of heterosexual couples to same-sex spouses as well as in campaigns for ‘rainbow families’ by the Ministry for Family, Senior Citizens, Women and Youth or LSVD that claim same-sex couples or ‘rainbow families’ *resemble heterosexual* family arrangements. This claim reinforces “the supposition that ‘equality’ requires ‘sameness’” (Richardson 2005: 519) and negates any heterogeneity among communities’ and people’s subject positions, resources, ways of living, and desires—and *makes them equal* to the dominant heterosexual norm and normality.

If the neoliberal transformation of the apparatus of sexuality is based upon emphasizing shared norms, it is obvious that integrating lesbians and gays stabilizes the assumingly shared norms and normalities (Raab 2011; Mesquita 2011). As many queer contributions have argued, integrating non-heterosexuals in the name of similarity and equality strengthens the classed, racialized and ability-centered normalities that structure heteronormativity. The neoliberalization of the apparatus of sexuality prolongs the “racial (e.g., Whiteness) and class (e.g., access to economic recourses) as well as sexual (e.g., monogamous, long-term and committed sexual relationship arrangements), gender (e.g., culturally appropriate performances of gender), and body normativities (e.g., economically productive body)” (Elia and Yep 2012: 885; see also Cooper 2004; Duggan 2002; Mesquita 2011; Puar 2007; Reddy 2011; Warner 1999).

Furthermore, the integration of lesbians and gays is based on desexualization, because it is only possible to frame lesbians and gays as same and equal to heterosexuals if sexuality is removed from the discourse. This desexualization is a prerequisite for transforming lesbians and gays into ‘normal citizens’ and is a mode of power that governs lesbians and gays “to adopt disciplined sexual practices through the internalization of the new norms of identity and sexual practices associated with a certain (heteronormative) lifestyle, with various rights granted through demonstrating a specific form of ‘domestic’ sexual coupledom” (Richardson 2005: 521). In a similar vein, Christine Klapeer demonstrates how the inclusion of lesbians as intelligible citizens with equal rights requires an “identification with the (heteronormative) status of a female citizen” and thus to consider her “‘sexual orientation’ as socially and politically ‘irrelevant’ and respectively her sexual difference” as ‘private’ (Klapeer 2014: 245, author’s translation). Such a form of integration leads to an “adaption of gender performance to the prevailing (heteronormative) gender norm” (Klapeer 2014: 245, author’s translation) and furthers the assumption that sexuality, intimacy, desire, relationships and family relations are ‘private’ issues.

Against this background I propose re-conceptualizing one of queer theory’s core notions. If we aim to fully grasp neoliberal sexual politics, it is necessary to recast heteronormativity as *heteronormalization*. In his lectures at the Collège de France in 1978 Foucault introduces a new understanding of how power operates through norms and normalization. He differentiates between three techniques of enacting power: normation, normativity, and normalization (Foucault 2007: 58). The law operates on the basis of an a priori given norm that distinguishes between what is allowed and what is forbidden—which Foucault describes as normativity. Disciplines also are based on an a priori given norm that classifies individuals. This normation also operates on the basis of a binary differentiation between ‘normal’ and ‘abnormal’. On the contrary, normalization does not operate based on an a priori given binary norm but through normality that is constituted in the process of governing. The technology of normalization aims “to reduce the most unfavorable, deviant normalities in relation to the normal, general curve, to bring them in line with this normal general curve” (Foucault 2007: 62). Normalization is neither built upon a norm/non-norm binary; nor is it an a priori given. Instead, it is a result of a form of governing that integrates certain deviations from the mean value. The “operation of normalization consists in establishing an interplay between these different distributions of normality and [in] acting to bring the most unfavorable in line with most favorable” (Foucault 2007: 63). Thus, the deviances are ‘immunized’ by integrating them (Lorey 2011).

For conceptualizing ‘heteronormativity’ in neoliberal societies, it follows that it not only operates as normativity but also as normalization. Heteronormativity not only functions through upholding the binary of homosexuality and heterosexuality but it also operates as *heteronormalization* through the normalizing integration of certain forms of non-heterosexuality. Heteronormalization is not built upon a binary of given norms and deviances, but instead it produces normality by integrating (some of) its deviances. In other words, it ‘immunizes’ (Lorey 2011) difference by integrating it. The normalizing integration of certain forms of homosexuality is thus also part of heteronormalization.

## Desiring an Anti-democratic, Violent State

Let me finally focus on the question how heteronormalization also helps to legitimate neoliberal statehood and its inherent anti-democratic and violent logics. In my argumentation, I refer to Foucault’s idea that the modern Western state is not an a-historical given, but rather an effect of social practices and of a desire for the state. Against this background, I argue that sexual politics are crucial for inciting such a desire for the state.

To be clear, my argument is not that neoliberalism is the first time in history when statehood is markedly anti-democratic and violent. On the contrary, as many scholars have argued, the Western state has always been violent and its promise of democracy has never been fully realized—in particular not for women, non-heterosexuals, migrants, indigenous populations, slaves, and all racialized others—which is why the anti-democratic and violent aspects of neoliberal statehood need to be understood as part of a historical continuum. The rise of neoliberal governmentality has led to a rearrangement of these anti-democratic and violent elements. The intrinsic aim of neoliberal governmentality to expand the logic of the market to cover all aspects of society means that techniques that were previously reserved for those who did not count as full citizens (women, non-heterosexuals, colonized people, indigenous populations, slaves, racialized others, migrants) have become more generalized and dispersed. Second, the discursive logic that accompanies anti-democratic and violent politics has also changed insofar that neoliberal governmentality aims to legitimate these politics by framing them as politics of freedom. Finally, the role of sexual politics in legitimating these politics has also been rearranged: sexual politics that promise to integrate some non-heterosexual ways of living are now being deployed to legitimate the neoliberal anti-democratic and violent state as I will show.

As argued above, the neoliberal flexibilization of the apparatus of sexuality entails the logic of privatization: Expanding state recognition to same-sex partnerships and families also fosters the idea that both care and sexuality are private issues. In this way, sexual politics support neoliberal technologies of power that depoliticize social issues by privatizing them. The neoliberal “expansion of a right to sexual privacy” (Duggan 2002: 180) calls for self-initiated activities where subjects govern themselves as *homines oeconomici* who desire to defend their individual right to freedom rather than enabling them to envision themselves as collective political agents. This supports a form of statehood that narrows the political in the name of individual freedom and self-determination—a form of statehood that can be described as anti-democratic as Wendy Brown has highlighted (2005, 2006). Following Lisa Duggan’s description of neoliberalism that “[t]here is no vision of a collective, democratic public culture, or of an ongoing engagement with contentious cantankerous queer politics” (Duggan 2002: 62) it can be argued that this helps to enable a state that acts anti-democratically because it reduces the scope of what is considered as politically negotiable in the name of freedom and self-determination. The “political sedative – we get marriage and military, then we go home and cook dinner, forever” (Duggan 2002: 62) operates as a technology of power that helps to (re-)produce an anti-democratic form of statehood.

The neoliberalization of the apparatus of sexuality also helps constitute, secure and strengthen a form of statehood that entails a form of “privatized social policy” (Foucault 2008: 145) that Johanna Oksala has—by expanding Foucault’s analysis of neoliberalism—described as violent form of social politics (Oksala 2011). Neoliberal social policy should not—as was the case (at least to a certain extent) in particular for non-migrant male citizens in the Fordist welfare state—compensate for the destructive forces of the market, but rather “intervene on society” (Foucault 2008: 145) so that “its objective will become possible, that is to say, a general regulation of society by the market” (Foucault 2008: 145). Privatizing social risks and exposing humans to the devastating consequences of the market—for instance, by cutbacks in health care—is what makes neoliberal governmentality particularly violent (Oksala 2011: 479). The violence of the neoliberal state prolongs material inequality and it does not protect people from structural risks but instead exposes people to these risks in an unequal manner. Sexual politics which claim ‘rainbow families’ are like heterosexual families, such as the LSVD campaign “It’s no different in our homes” (“Bei uns geht’s auch nicht anders zu”), supported by the City of Berlin, help to uphold a state with a violent social policy. State recognition of same-sex partnerships expands the group of people the state addresses to take responsibility and compensate for social risks in a private manner like heterosexual families. Therefore, arguing for the integration of lesbians and gays into the realm of state-protected family formations not only plays into the privatization of social reproduction, but allows the neoliberal state to outsource social services and pursue a violent social policy where individuals are responsible for compensating for the consequences of the market-based structure of society. The promises of individual freedom and sexual diversity that accompany the neoliberalization of the apparatus of sexuality act as mode of governing that incite a will and desire for a state that relies on a form of governmentality that pursues the privatization of social risk and the violent exposure of subjects to the devastating consequences of the market.

Furthermore, the flexibilization of the apparatus of sexuality aids in affirming the violent side of neoliberal statehood, because practices that appreciate the neoliberal state’s ‘modern’ and ‘democratic’ homo-tolerance also support its violent biopolitics. In the campaign “Berlin advocates Self-Determination and Acceptance of Sexual Diversity” one of the key measures for overcoming homophobia is to foster a “dialogue” (Senate of Berlin 2010: 6, author’s translation) with NGOs and other civil society institutions. The campaign literature highlights that especially “interreligious and other initiatives dealing with integration politics that support the acceptance of sexual diversity should be taken into consideration” (Senate of Berlin 2010: 6, author’s translation). These dialogues should help to overcome homophobia and are explicitly framed as part of a democratization process (Senate of Berlin 2010: 6: 26). We find a similar logic in a press release that LSVD sent out in February 2016. The press release entitled “Integration does not come by its own” (“Integration kommt nicht von allein”) (LSVD 2016) demanded that ‘sexual diversity’ should become part of the curriculum of integration courses for migrants and that “[a]ll integration programs and language learning materials should be conceived in a way that teaches and fosters democracy, diversity and individual rights to freedom” (LSVD 2016, author’s translation). To include “the realities of the everyday lives of LGBTI persons in the curriculum and discussing them in a way that builds respect” is viewed as “the government’s responsibility” (LSVD 2016, author’s translation).

These two examples show that within neoliberal sexual politics, sexual tolerance, diversity and openness are framed as hallmarks of democracy. At the same time, these discourses construct (groups of) people of ‘other’ religions and nationalities as target groups who need special education to reach a state where they can become tolerant, open and truly democratic. Migrants—and in particular Muslim migrants—and Muslim Germans are constructed as potentially homophobic and in need of education regarding sexual diversity, which is simultaneously framed as education in democracy. This “‘migrant homophobia’ discourse” (Haritaworn 2010: 75)—that has also been discussed in left and liberal newspapers from 2008 as Haritaworn highlights (2010; 2015)—led to a number of campaigns throughout the last years. For instance, the City of Berlin funded a poster campaign by LSVD entitled “Show respect” (“Zeig Respekt”) that was published in German, Turkish and Arabic. Employing the same discursive logic, LSVD and MANEO, the gay anti-violence hotline in Berlin, initiated same-sex ‘kiss-ins’ at “symbolic places” that are “not an easy place for open gays, lesbians and transgenders” (MANEO 2016) such as districts with a high number of migrant population (Haritaworn 2015: 100).

These racialized and nationalist sexual politics not only support the continuation of a racialized, neocolonial and nationalist distinction between an imagination of Germany and Western Europe as progressive, liberal and democratic in contrast to non-Western ‘Others’ as not-yet modern and democratic (Ferguson and Hong 2012; El-Tayeb 2011; Haritaworn 2015; Puar 2007). They also help to advance a desire for a state that is built upon neocolonial and racializing logics. In other words, the flexibilization of the apparatus of sexuality as a hallmark of modernity, democracy and civilization functions as a technology that renders the neoliberal state desirable. The flexibilization of sexual politics incites a desire to belong to a homonormative and homonational state because that also means that one belongs to a tolerant, democratic and modern national community. Therefore, in response to Chandan Reddy’s question “why national norms might desire GLBTQ desire for formal equality” (Reddy 2011: 193), I argue this is because the normalizing integration of certain kinds of lesbian and gay lifestyles initiates a desire based on a (neo-)colonial and racialized construction of ‘modernity’ but—because it is part of the neoliberal governmentality—it is no longer based upon rigid heteronormativity but on flexible heteronormalization.

The flexibilization of the apparatus of sexuality means that lesbians and gays as “‘ordinary’, ‘*normal*’ citizens” (Richardson 2005: 519) have become part of the population whose lives should be optimized and proliferated whereas at the same time certain groups of people are rendered as ‘disposable’—especially illegalized migrants (El-Tayeb 2011; Puar 2007; Reddy 2011; Richardson 2005). The same state that promises to protect heteronormalized lesbians and gays continues to construct other groups within the population—non-white, Muslim, migrant and/or illegalized people—as dangerous and deviant (El-Tayeb 2011; Eng 2003; Haritaworn 2015; Puar 2007). This logic can also be found in LSVD’s press release, which not only reproduces a we-they logic in which ‘liberal Germans’ are opposed to ‘not-yet liberal migrants’ in need of ‘integration courses’ but also fails to relate struggles of sexual politics to struggles against the global neo-colonial migration regime.

Thus, neoliberal homo-tolerant sexual politics help to prolong a violent dynamic of biopower. As Foucault (1997) has argued, the integration of some groups of the population into the realm of those whose lives should be optimized and deserve protection requires the ‘killing’ of others, whereas Foucault deploys a broad understanding of ‘killing’ here: “the fact of exposing someone to death, increasing the risk of death for some people, or, quite simply, political death, expulsion, rejection, and so on” (Foucault 1997: 256). These biopolitical dynamics have shifted with neoliberalism; they have not become a thing of the past, but through the use of sexual politics, they are prolonged in the present.

Finally, the neoliberalization of the apparatus of sexuality helps to affirm the anti-democratic and violent claim of the neoliberal governmentality that there is no alternative to it. Oksala argues that the aim of neoliberal governmentality to make any other form of organizing politics, society, the public, social relations, working relations and self-relations impossible, unthinkable and unlivable is violent and can only be realized by violent means (Oksala 2011: 479). Here, also heteronormalizing policies help to push this aim further: As argued above, integrating certain gay and lesbian lifestyles into the continuum of normality can be interpreted as ‘immunizing normalization’ (Lorey 2011), because it not only normalizes the formerly ‘othered’ but also immunizes any alternatives to the existing social and political order. As shown above, the immunizing normalization is embedded in a neocolonial, nationalist and racializing logic. The immunizing normalization that configures some lesbians and gays as a protectable part of the population—those who resemble the white, middle class, non-Muslim heterosexual citizens—rests upon the construction of a racionalized ‘Other’—‘German-Turkish people’, ‘Muslim migrants’—from whom the white, non-Muslim, German lesbians and gays who can be integrated into the homonational normality need to be protected (see also Haritaworn 2015). This nationalist, neo-colonial, racializing demarcation line constructed between those who can and should be integrated into the national normalized ‘we’ (‘from within’) and those who are configured as a (threatening) ‘others’ operates as technology of power that helps to strengthen a form of governmentality that aims to omit any alternatives to the existing social and political order. It fosters neoliberal governmentality’s ambition making every alternative for organizing society and social relations unthinkable and unlivable.

## Conclusion: Dangerous Entanglements

I agree with Duggan’s description of neoliberal sexual politics that

“[t]his new homonormativity comes equipped with a rhetorical recoding of key terms on the history of gay politics: ‘equality’ becomes narrow, formal access to a few conservatizing institutions, ‘freedom’ becomes impunity for bigotry and vast inequalities in commercial life and civil society, the ‘right to privacy’ becomes domestic confinement, and democratic politics itself becomes something to be escaped” (Duggan 2003: 66).

However, I want to add that neoliberalism does not only have an impact on sexual politics; the flexibilization of the apparatus of sexuality also advances neoliberal governmentality and neoliberal statehood and is therefore intrinsic to neoliberal governmentality. Neoliberalism is just as anti-social, anti-democratic and violent, as it is tolerant, flexible and pluralistic—not only are the former its hallmarks, the latter are too. Neoliberalism deploys the promise of tolerance, flexibility and pluralism in order to fulfill its anti-social, anti-democratic and violent agenda. The neoliberalization of sexual politics creates new forms of old power relations, which make subjects governable as sexualized subjects, incite a desire to a violent and anti-democratic state, and put nations, populations and subjects in unequal positions through employing a racialized and neocolonial matrix.

In light of her diagnoses that neoliberalism is intrinsically anti-democratic (and we could add: also violent), Wendy Brown raises the question of “how much legitimacy neoliberal governance requires from a democratic vocabulary” (Brown 2005: 49)? As I have argued, despite its anti-democratic and violent elements, neoliberal governmentality does require the consensus and acceptance of the majority of the population—not least because neoliberalism is not and cannot be forced upon the population, as it is governed and therefore also relies on the subjects’ self-activation. Thus, neoliberalism also needs to be investigated as political project that engages people, deploys their hopes and promise them a good life, more freedom, wealth or personal fulfillment. Sexual politics need to be investigated as technologies of power that help to organize acceptance and consensus within neoliberalism.

What are the consequences of this diagnosis of neoliberalism as embedded in homonormative and homonational politics, which help to incite an anti-democratic and violent state in political terms? Let me conclude with two thoughts. First, what follows from the entanglement of statehood with sexual politics as analyzed above, is that conceiving the state as a protector or guarantor of security limits queer politics’ emancipatory capacity because, as Foucault has taught us, the will and desire to address the state is already an effect of power. Given that the agenda of privatization and erasing all forms of alternatives contribute to the violence of the neoliberal state, queer politics that deploy ‘individual freedom’ and the ‘right to privacy’ also comply with neoliberal politics. Instead of struggling for the inclusion of some, queer emancipatory politics need to search for and invent new, different collective forms of organizing society, social relations, self-relations, care, kinship, and economy.

Second, the analysis shows that emancipatory queer politics cannot be single-issue-politics—because sexual politics are always entangled with nationalist, racializing and capitalist projects and are a productive element in constituting them. Consequently, queer politics that aim to be emancipatory for everyone must address racialized, nationalist and capitalist biopolitics on a global scale. What Chandan Reddy problematizes regarding the struggles for obtaining recognition for same-sex partnerships in the USA also applies to the European and German contexts: Reddy critiques that these struggles are entirely disconnected from other social struggles such as those of (illegalized) migrants against the neo-colonial regime of migration (Reddy 2011). Reddy points out the paradox of struggles that focus on same-sex issues, which demand the realization of the promises of modernity—but at the same time these promises are only applied to people whose nationality is ‘proper’ because (illegalized) migrants were not viewed as part of these struggles in the first place. As long as queer struggles fail to address sexualized, racialized, capitalist, neo-colonial biopolitics on a larger scale, the dynamics that Foucault has described as crucial for modern Western biopolitics in a capitalist society cannot be overcome: a dynamics that not only divides humans into a group that is seen as worth of protection and a group that is framed as ‘disposable’ but also a dynamic where the ‘good life’ of the former requires the (social) death of the latter.

## Footnotes

1 Clearly, Berlin has been a site where queer sexualities came together and proliferated long before the city started to present itself as ‘rainbow city’ as part of a neoliberal political strategy. The following argumentation aims to make visible that the framing of Berlin as ‘rainbow city’ can be read as an example how neoliberalism also gains power by selectively incorporating and assimilating elements of queer (subversive) history. This strategy of selectively incorporating former (more) radical or subversive practices can be viewed as governing technology that helps to render neoliberalism desirable.
